# Ischemic Stroke Induces ROS Accumulation, Maladaptive Mitophagy, and Neuronal Apoptosis in Minipigs

**DOI:** 10.4014/jmb.2409.09003

**Published:** 2024-11-14

**Authors:** Jie Chen, Yanan Bie, Yajin Guan, Wen Liu, Fei Xu, Tianping Liu, Zilong Meng, Mengqi Gao, Jiawei Liu, Shuilin Xie, Weiwang Gu

**Affiliations:** 1 Guangdong Provincial Key Laboratory of Large Animal Models for Biomedicine, South China Institute of Large Animal Models for Biomedicine, School of Pharmacy and Food Engineering, Wuyi University, Jiangmen 529000, P.R. China; 2 School of Life Sciences and Biopharmaceutics, Guangdong Pharmaceutical University, Guangzhou 510000, P.R. China; 3 Institute of Comparative Medicine & Laboratory Animal Management Center, Southern Medical University, Guangzhou 510000, P.R. China; 4 Guangdong Mingzhu Biotechnology Co., Ltd., Foshan 528000, P.R. China; 5 Institute of Neuroscience, Department of Neurosurgery the Second Affiliated Hospital of Guangzhou Medical University, Guangzhou 510000, P.R. China; 6 GuangDong 999 Brain Hospital, Guangzhou 510000, P.R. China; 7 School of Biology and Biological Engineering, South China University of Technology, Guangzhou 510006, P.R. China

**Keywords:** Reactive oxygen species accumulation, maladaptive mitophagy, neuronal apoptosis, minipig, acute ischemic stroke

## Abstract

Reactive oxygen species (ROS)-induced adaptive/maladaptive mitophagy plays an essential role in the pathophysiology of acute ischemic stroke (AIS). However, most studies have been conducted using rodent models, which limits their clinical application. In this study, we aimed to develop porcine models of permanent stroke and observe the pathophysiological alterations caused by acute ischemic stroke, focusing on ROS-induced mitophagy. Miniature pigs were subjected to lateral frontotemporal electrocoagulation, which resulted in permanent middle cerebral artery occlusion. We investigated global brain damage and mechanisms of adaptive/maladaptive mitophagy caused by ROS and global brain inflammation after AIS. An early neuroinflammatory response was observed in the ipsilateral hemisphere. ROS levels were significantly elevated in the ipsilateral hemisphere and slightly elevated in the contralateral hemisphere. ROS accumulation may be attributed to the increased production and impaired elimination of ROS. In addition, mitophagy and apoptosis were detected in the ischemic core, which may be attributed to ROS accumulation. We propose "distinct-area targeting” interventions aimed at maladaptive mitophagy within the ischemic core of the infarct hemisphere, which may provide new therapeutic targets for the treatment of AIS.

## Introduction

Acute ischemic stroke (AIS) is the second leading cause of death globally, and usually leads to long-term disability in surviving patients. AIS poses a considerable socioeconomic burden worldwide [1]. Although remarkable research efforts have been devoted to developing AIS regimens, only a few approaches have demonstrated efficacy in reducing mortality and disability. In addition, new drugs and therapeutic strategies have shown efficacy in preclinical studies (the vast majority of which were tested in rodents), but have not achieved the primary endpoints in large clinical trials in humans. This discrepancy can be attributed to notable anatomical and physiological differences between rodent and human brains.

The minipig is an alternative and advanced animal model for AIS because the porcine brain anatomy is more similar to the human brain than the rodent brain. For instance, the porcine brain is gyrencephalic and has a white-matter-to-gray-matter ratio comparable to that of the human brain [2]. Therefore, the minipig model can be used to recapitulate the pathophysiology of AIS more accurately, thereby serving as a useful tool for developing treatment approaches.

After AIS, primary brain injury is triggered by several detrimental events, including oxidative stress, mitochondrial disturbance, and global neuroinflammation. These events can further promote secondary brain injury [3]. Conventional prognostic risk factors, such as advanced age and hypertension, fail to fully explain the poor clinical outcomes in patients with ischemic stroke. Recently, mitophagy has been reported to play a role in secondary brain injury following ischemic stroke. In particular, mitophagy appears to be critical for eliminating dysfunctional/superfluous mitochondria [4]. Furthermore, oxidative stress triggers and fuels mitophagy after I/R injury [5]. “Adaptive mitophagy” may be a protective response in neuronal cells in physiological conditions and under moderate stress, whereas “maladaptive (dysfunctional) mitophagy” might lead to cell death under overwhelming stress [6-9]. Currently, the role of mitophagy in AIS and its relationship with neuronal apoptosis remains largely elusive, especially in large animal models [10]. Therefore, in this study, we aimed to investigate pathophysiological alterations caused by AIS in a porcine model by focusing on adaptive/maladaptive mitophagy.

## Materials and Methods

### Animals

We used 4-month-old male Tibetan minipigs weighing 25 kg (Guangdong Mingzhu Biotechnology Co., Ltd., China). Minipigs were randomly assigned to experimental groups. All pigs were housed under controlled temperature, humidity, and light (12:12 h light/dark cycle) conditions with *ad libitum* access to food and water.

### General Surgical Preparation

Twelve pigs were randomly divided into two groups. Six animals received permanent middle cerebral artery occlusion (MCAO) and six received a sham operation. The pigs were initially sedated with an intramuscular injection of 2 mg/kg ketamine hydrochloride (Shenda, China) and 3 mg/kg zoletil (Virbac, France). They were then intubated and artificially ventilated with 1.5%–2% isoflurane (Kimura, Japan) in a nitrous oxide/oxygen mixture. A portable plug-in monitor (Q5; Biolight, China) was used for continuous physiological monitoring of oxygen saturation, heart rate, and respiratory rate. The pigs maintained a normal heart rate (100 beats/min) and a normal respiratory rate (18 breaths/min). Moreover, they were adequately oxygenated (PO_2_: 100 mmHg) while in a state of anesthesia-induced unconsciousness. During the operation, a heating blanket was used to maintain body temperature at 38°C.

### MCAO by Electrocoagulation and Transection

**Fig. 1:**
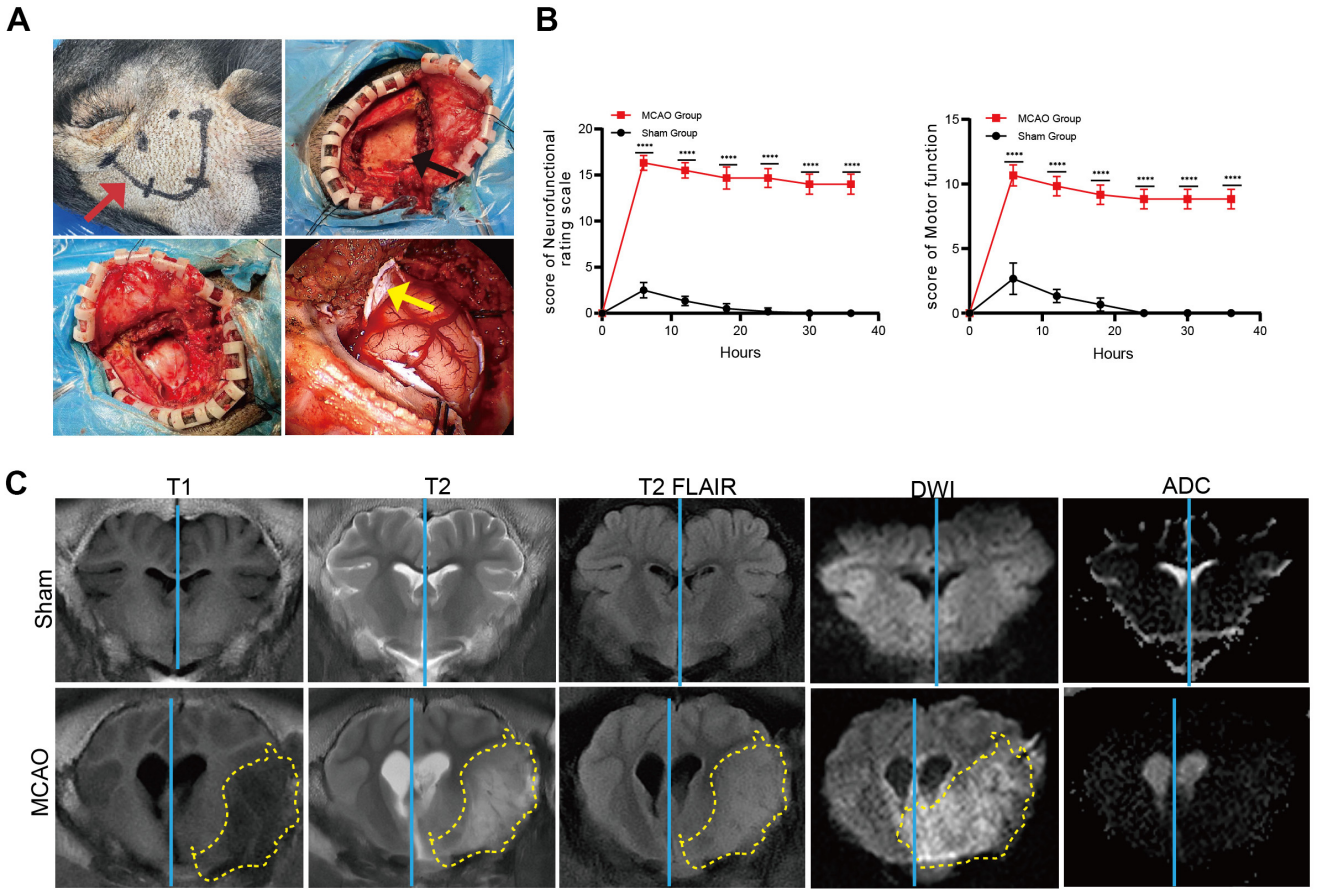
Neurological status scores and MRI scans to validate ischemic brain lesions after AIS. (**A**) From left to right: Marking positioning (red arrow); scalp flaps were created to expose the skull (the skull is marked by a black arrow); craniotomy was performed using a skull drill; dural opening and electrocoagulation operation (the dura is marked by a yellow arrow). (**B**) Evaluation of the neurofunctional rating scale and motor function within 36 h postoperatively (*n* = 6). (**C**) Representative MRI scan images of the porcine brain. T1, T2, T2 FLAIR, DWI, and ADC imaging showed midline shift (blue line) or cytotoxic edema (yellow dotted area), respectively (*n* = 6). Data are presented as the mean ± SD. Student’s *t*-test (**B**) was performed; *p < 0.05; **p < 0.01; ***p < 0.001; and ****p < 0.0001.

The pigs were subjected to frontotemporal orbital rim osteotomy in the lateral position to induce permanent MCAO, as previously described [11]. Briefly, an entry burr hole was created at the anterior portion of the superior temporal line and expanded to perform frontotemporal craniectomy (Fig. 1A). The dura mater, arachnoid cisterns, and basal cisterns were opened sequentially. The internal carotid artery, posterior communicating artery, and optic nerve were identified, and the terminal carotid artery and middle cerebral arteries were exposed. Ischemia was induced by electrocoagulation of the artery M1 (1.5 mm) from a point proximal to the origin of the lenticulostriate artery. The artery was then transected to ensure complete occlusion. The sham group was treated following the same procedure but without artery M1 electrocoagulation and transection.

### Post-Operation Management

The animals were allowed to awaken after extubation. The pigs were subjected to continuous physiological monitoring until their body temperature, heart rate, and respiratory rate returned to normal (Table 1). Ketamine (10 mg/kg) was administered for 1 h to manage perioperative pain if required [12]. The animals were injected intramuscularly with 1.5 million units of penicillin and streptomycin to prevent infection. Mannitol (30 ml, 1 g/kg) was administered intravenously to prevent brain edema [12].

### Porcine Magnetic Resonance Imaging (MRI)

The pigs were subjected to MRI 36 h after the stroke was induced to confirm ischemic stroke and determine the infarct volume and pattern. Under general anesthesia, the pigs were placed in a supine position on a 3.0 Tesla MRI system (uMR 790). Standard multiplanar brain MRI series were acquired in the coronal plane. T1 imaging was performed (repetition time [TR]: 2100 ms; echo time [TE]: 13.66 ms; bandwidth: 180 Hz/pixel; echo interval: 13.66 ms; phase acceleration factor: 2; slice thickness: 5 mm), followed by T2 fluid-attenuated inversion recovery (FLAIR) imaging (TR: 8000 ms; TE: 105 ms; bandwidth: 300 Hz/pixel; echo interval: 10.5 ms; phase acceleration factor: 2; slice thickness: 5 mm), and T2 imaging (TR: 5000 ms; TE: 110.88 ms; bandwidth: 180 Hz/pixel; echo interval: 15.84 ms; phase acceleration factor: 2; slice thickness 5 mm). Diffusion-weighted imaging (DWI) was performed using a spin echo-plane sequence (TR: 2500 ms; TE: 95 ms; bandwidth 640 Hz/pixel; echo interval: 1.27 ms; phase acceleration factor: 3).

### Evaluation of Neurological Status

Every 6 h after MCAO, the neurological status of each animal was evaluated according to the neurological examination grading scale for minipigs (Tables S1 and S2), which included scores generated from the neurofunctional rating scale and the motor functional assessment [13]. The neurofunctional rating scale assessed appetite (4 points), standing position (5), head position (2), utterance (2), and gait (3). Motor function assessment included the assessment of the motor deficit scores of the forelimbs (4), hind limbs (4), and facial paresis (1).

### Brain Sample Acquisition

MRI images were used as a guide for sample selection. Brain samples of the ischemic ipsilateral hemisphere on the right and the contralateral hemisphere on the left sides were acquired for further analysis.

### Histological Analysis

Brain samples were fixed with 4% paraformaldehyde and embedded in paraffin. Microsections (5-mm thick) were prepared for hematoxylin and eosin (H&E) staining, immunohistochemistry, or immunofluorescence staining, which was performed as previously described [14, 15]. Images were visualized using a Nikon A1 confocal microscope (Nikon, China). The primary and secondary antibodies used are listed in Table S3.

### TUNEL Staining

TUNEL staining was performed on paraffin-embedded samples using a TUNEL Assay Kit (#64936; Cell Signaling Technology) according to the manufacturer’s instructions. Images were obtained using an OLYMPUS BX61 microscope (OLYMPUS, Japan).

### LC-MS Metabolomics Processing and Metabolomics Data Analysis

Forty brain samples were analyzed using an LC-MS platform (Ultimate 3000LC, Q Exactive; Thermo Fisher Scientific). Samples were divided into four groups: MCAOR (the infarcted ipsilateral hemisphere of the MCAO pigs), MCAOL (the contralateral hemisphere of the MCAO pigs), ShamR (the right hemisphere of the sham pigs), and ShamL (the left hemisphere of the sham pigs). LC-MS metabolomics processing was performed at Majorbio Co., Ltd. (China) as previously described [16]. The metabolomics data were transformed and analyzed using a self-built database on the Majorbio I-Sanger Cloud Platform (https://www.i-sanger.com). Orthogonal partial least squares discriminant analysis and Student’s *t*-tests were used to identify the metabolic alterations among experimental groups, and *p*-values < 0.05 were considered to indicate statistical significance. The Majorbio I-Sanger Cloud Platform (https://www.i-sanger.com) was used to characterize the screened differential metabolites. Significantly differential metabolites between groups were analyzed for expression pattern clustering using the gplots package in R (version 1.6.2). The impacts of AIS on metabolic pathways and metabolite set enrichment were analyzed using the Stats package in R (version 1.6.2) and SciPy (version 1.0.0) in PYTHON.

### Transmission Electron Microscopy (TEM)

Fresh tissues, trimmed to approximately 1 mm × 1 mm × 1 mm, were embedded in 2.5% glutaraldehyde at 4°C overnight. The specimens were fixed in veronal acetate (pH 7.3) containing 1% osmium tetroxide for 1 h, dehydrated with ethanol, and embedded in Epon. Then, 1 μm-thick sections were counterstained with uranyl acetate and lead citrate and visualized with a HITACHI HT7700 electron microscope.

### Enzyme-Linked Immunosorbent Assay (ELISA)

Samples were snap-frozen in liquid nitrogen. Then, a 100-mg piece of tissue was added to 1 ml of phosphate-buffered saline (pH 7.4) in a tube. Next, the tissue was homogenized using a grinder (60 Hz) and centrifuged for 20 min at 3000 g at 4°C. The supernatant was collected and stored at −80°C. ELISA was performed according to the manufacturer’s instructions. Absorbance was measured at 450 nm using a microplate reader. The concentrations were calculated according to the standard curves. The ELISA kits used are listed in Table S3.

### Western Blotting Analysis

Samples were homogenized in radioimmunoprecipitation assay lysis buffer. Proteins were separated via 6–15%sodium dodecyl-sulfate polyacrylamide gel electrophoresis and transferred to a polyvinylidene difluoride membrane (Millipore, USA). The membrane was incubated with primary antibodies overnight at 4°C, followed by incubation with a horseradish-peroxidase-labeled goat or mouse anti-rabbit immunoglobulin G for 1 h. GAPDH or β-actin was used as a control. The primary and secondary antibodies used are listed in Table S3.

### Assessment of Oxidative Stress

Samples were homogenized, and the concentrations of cellular reactive oxygen species (ROS) and MMP-9 were detected using ELISA kits. Superoxide anion (O_2_^–^) levels were measured using a Micro Superoxide Anion Assay Kit; the fluorescence of the contents was measured at 530 nm. H_2_O_2_ was measured using a kit (BC3595; Solarbio); the fluorescence of the contents was measured at 415 nm. Malondialdehyde (MDA), superoxide dismutase (SOD), catalase (CAT), and glutathione peroxidase (GSH-PX) in the tissues were quantified using commercial kits according to the manufacturer’s instructions. NAD^+^, NADH, and NAD^+^/NADH were quantified according to the manufacturer’s instructions using an NAD^+^/NADH Assay Kit with water-soluble tetrazolium 8. The kits used are listed in Table S3.

### Quantification and Statistical Analysis

Each experiment was repeated three times independently. We used the G*power analysis program [17] to calculate the minimal number of animals required to detect any significant effect in MCAO models. According to our previous studies in pig models [14], the sample size for the two-group *t*-test was six per group, with a G*power of 0.85 and α = 0.05, which is a well-accepted power value for statistical calculation. Data are presented as the mean± standard deviation and were analyzed using SPSS version 20.0 (IBM, USA) and GraphPad Prism version 9.0 (GraphPad Software, USA). Statistical significance was determined using the Student’s *t*-test or one-way analysis of variance with Tukey’s post hoc test to compare differences between groups. All tests were considered statistically significant at *p* < 0.05.

Forty brain samples were analyzed using the LC-MS platform. The metabolomics statistical data were analyzed using the free online Majorbio I-Sanger Cloud Platform (https://www.i-sanger.com), distance calculation algorithms, such as Spearman’s for samples, Pearson’s for metabolites, and the clustering method for H clusters as previously described [16]. *P*-values were adjusted using the false discovery rate (FDR), and the corrected *P*-values below 0.05 were regarded as statistically significant.

Quantification of TUNEL-stained slides and sections for TEM was performed using semi-quantitative analyses. For TUNEL samples, ten random, non-overlapping fields (magnification, ×200) of brain tissue from each specimen were photographed and pictures were automatedly single-cell counted for green fluorescence using ImageJ software. For TEM, 10 random, non-overlapping fields (magnification, ×10,000) of brain tissue from each specimen were counted by analysts in a double-blind manner. In all fields, the mean counts were averaged to yield the final score for each specimen.

### Ethical Approval

All experiments were approved by the Animal Committee of Guangzhou Huateng Biomedical Technology Co., Ltd. (approval number HTSW220202) and conducted in compliance with the Guide for the Care and Use of Laboratory Animals [18].

## Results

### Minipigs Displayed Severe Hemiplegia 36 h after MCAO

Throughout the operation period, heart rate, core body temperature, respiratory rate, and oxygen saturation remained stable at baseline levels (Table 1). All animals survived for 36 h after surgery until the endpoint. Loss of arterial pulsation, due to electrocoagulation and transection of the MCAO was easily observed using an operating microscope. Six hours after surgery, the neurological status of the pigs was evaluated. The pigs in the MCAO group displayed loss of appetite and hemiparesis in both the forelimbs and hindlimbs, and were unable to raise their heads. No abnormal behavior was observed in the sham-operated group. Neurological status was scored as early as 6 h after AIS. The total deficit and motor function scores of the MCAO group were significantly higher than those of the sham group at 36 h. Indeed, the scores in the sham group decreased to nearly zero (Fig. 1B).

### MRI Scan to Validate Ischemic Brain Lesion after AIS

An MRI scan was performed 36 h after AIS to evaluate ischemic lesions and perfusion of the brain. In all MCAO models, infarcts were noticeable and mainly located in the basal ganglion, internal capsule, and cortex, which are all MCA-supplied areas (Fig. 1C). In T1 and T2 sequences, we observed a shift of the midline to the contralateral hemisphere and compression and distortion of the parenchyma and ventricles. The high-intensity area in T2 and T2-FLAIR sequences indicated brain edema in the vascular-occluded hemisphere. Hyperintensity in DWI and ADC suggested ischemic features in the ipsilateral hemisphere (Fig. 1C). The bilateral cerebral hemispheres were symmetrical in the sham group, and no signs of abnormal signals in the sulci, fissures, or cisterns were detected, suggesting that no brain damage had occurred.

### Histology of Brain Edema and Cellular Necrosis after Ischemic Stroke

**Fig. 2:**
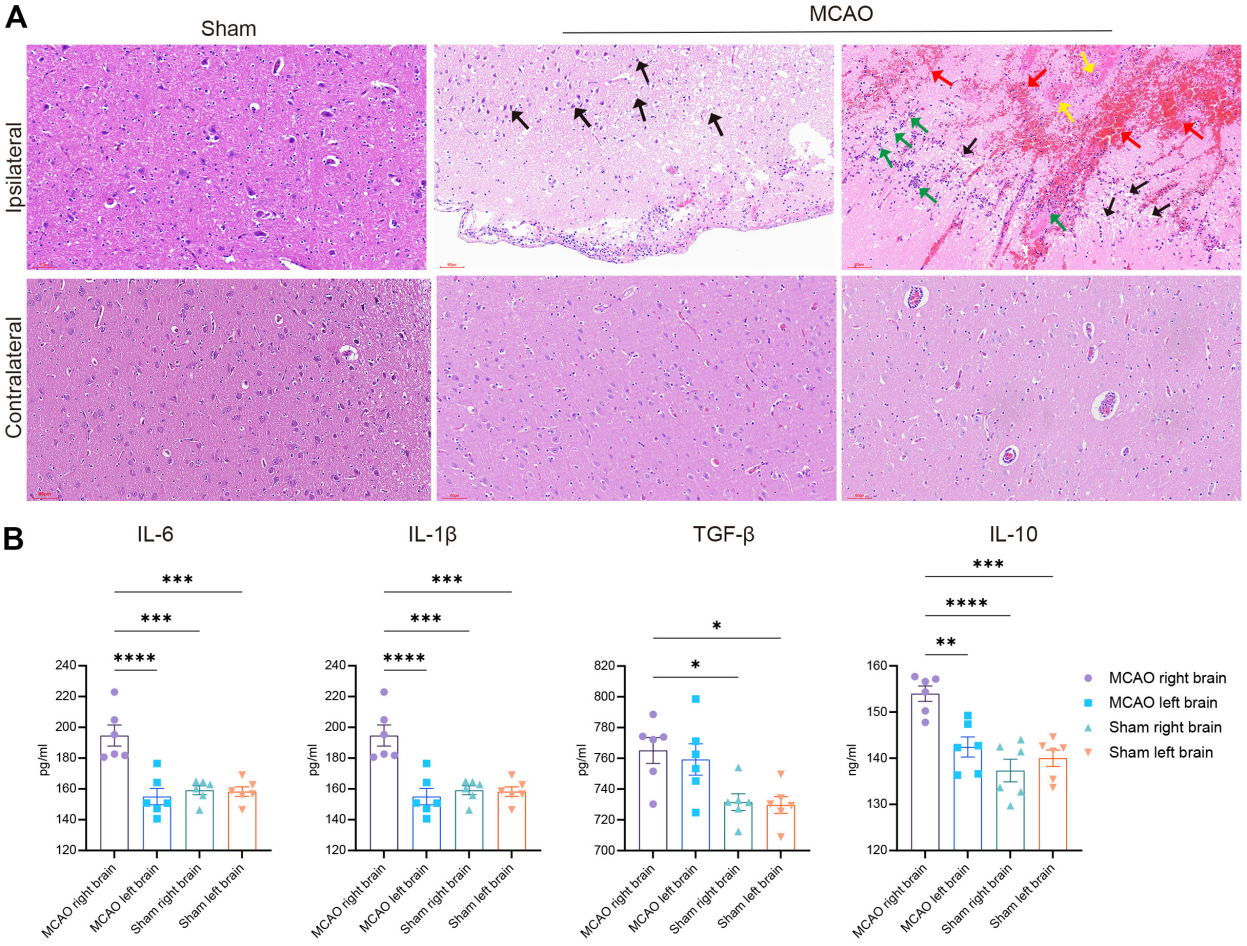
Histology of brain edema, cellular necrosis, and the early neuroinflammatory response after ischemic stroke. (**A**) H&E staining of ipsilateral (right) and contralateral (left) sides of brain tissues from sham and MCAO groups. The tissue was locally damaged, softened, and structurally sparse with a large number of necrotic and lysed microglia. More cavity formation was observed (black arrow) along with multiple hemorrhages and a large number of erythrocytes (red arrows). A large number of blood vessel walls were necrotic, with eosinophilic homogeneity (yellow arrows), and a large number of inflammatory cell infiltrations were also observed at the site of the injury (green arrows). Scale bar = 60 μm; *n* = 6. (**B**) ELISA analysis of the protein levels of cellular inflammatory factors, including IL-6, IL-1β, TGF-β, and IL-10 (*n* = 6). Data are presented as the mean ± SD. Sham group versus MCAO group; *n* = 6 per group. One-way ANOVA and Tukey’s multiplecomparisons pos*t*-test were performed; *p < 0.05; **p < 0.01; ***p < 0.001; and ****p < 0.0001.

In all MCAO groups, H&E staining of the infarcted ipsilateral hemisphere showed necrosis of neurons, as evidenced by the formation of a large number of cavities and cracks, accompanied by hemorrhage and vascular dilation (Fig. 2A). Many microglia infiltrated the peri-infarct areas, the vessel wall was necrotic, and the contralateral hemisphere exhibited vascular sleeves and limited microglial infiltration (Fig. 2A). The brain tissues in the sham group showed a compact structure and normal neuronal perikaryal, with uniform distribution and coloration.

### Early Neuroinflammation Response Was Observed in the Infarcted Hemisphere

Neuroinflammation represents one of the predominant pathophysiological processes for “secondary brain injury” after ischemic stroke wherein microglia act as the major cell population [19]. We examined the post-stroke inflammatory response in our model and found that the levels of pro-inflammatory cytokines, including interleukin (IL)-1β and IL-6, were significantly increased in the infarcted brain tissues (Fig. 2B). Notably, the levels of anti-inflammatory cytokines such as IL-10 and tumor necrosis factor-β were also upregulated in the infarcted hemisphere (Fig. 2B). These data suggest that AIS may trigger both pro- and anti-inflammatory responses in the brain after stroke and imply an intimate interaction between them.

### Metabolomic Profiling Revealed an Essential Role of ROS in Brain Injury after AIS

**Fig. 3:**
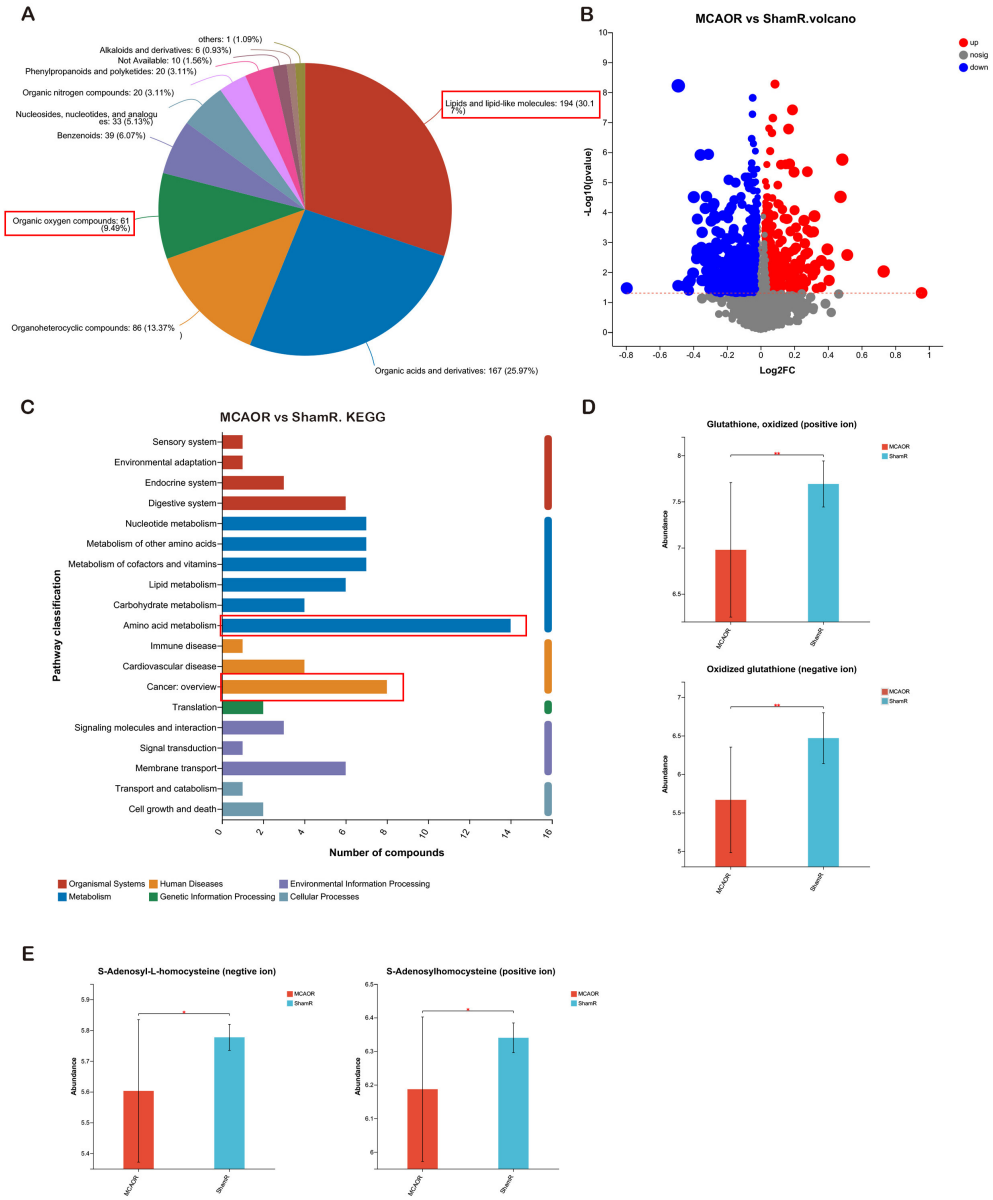
Metabolomic profiling revealed the essential role of ROS in brain injury after AIS. (**A**) Pie chart of the superclass of chemical taxonomy based on the HDMB database for the MCAOR, MCAOL, ShamR, and ShamL groups. (**B**) Volcano plots showing the magnitude and significance of metabolites (including positive and negative ions) in the MCAOR group compared with the ShamR group. (**C**) KEGG pathway enrichment of differential metabolites in the MCAOR group compared to the ShamR group. (**D, E**) The abundance of ROS-related metabolites (including positive and negative ions) in the MCAOR group compared that to in the ShamR group. MCAOR, the ipsilateral (right) sides of brain tissues from the MCAO group; MCAOL, the contralateral (left) sides of brain tissues from the MCAO group; ShamR, the right sides of brain tissues from the Sham group; ShamL, the left sides of brain tissues from the Sham group. Data are presented as the mean ± SD (*n* = 10). Student’s *t*-test (**D, E**) was performed; *p < 0.05; **p < 0.01.

Metabolic stresses, such as hypoxia and oxidative stress, in ischemic brains have been reported as detrimental post-stroke factors [20]. To gain a comprehensive perspective of the metabolomic alterations after AIS, we performed untargeted metabolomic analysis to reveal the differences between the ipsilateral and contralateral hemispheres from the MCAO and sham porcine groups. A total of 194 metabolites were included in the “lipids and lipid-like molecules” term, followed by “organic acids and derivatives” (*n* = 167), “organoheterocyclic compounds” (*n* = 86), and “organic oxygen compounds” (*n* = 61; Fig. 3A). There were 111 metabolites (66 positively ionized metabolites and 45 negatively ionized metabolites) that exhibited a two-fold change between the ipsilateral hemisphere of MCAO pigs (MCAOR) and the right hemisphere of sham pigs (ShamR; Fig. 3B). The differential metabolites identified were assigned to the KEGG database, which revealed 19 KEGG second-grade pathways. The top term was “amino acid metabolism,” followed by “cancer: overview” (Fig. 3C). We further found that metabolites involved in the “chemical carcinogenesis: ROS” pathway in the “cancer: overview” category, including glutathione disulfide and S-adenosyl-L-homocysteine, in the positive- and negative-ion modes, were significantly decreased in the MCAOR group (Fig. 3D and 3E). These data suggest that the ROS pathway plays a critical role in brain injury following AIS.

**Fig. 4:**
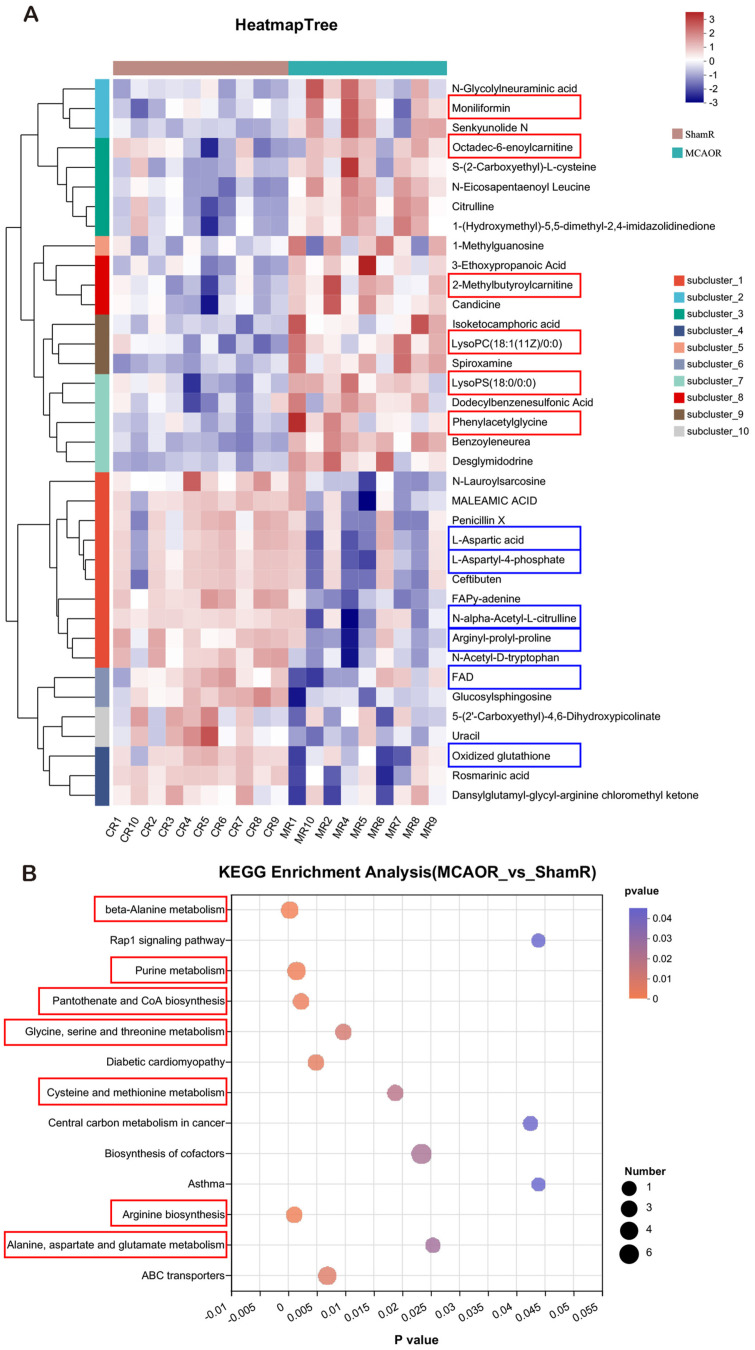
Metabolomic profiling revealed differential metabolites linked to the mitochondria after AIS. (**A**) Hierarchical clustering analysis for identification of different metabolites in brain tissues by comparing the MCAOR and ShamR groups. Each column represents a sample, each row represents a metabolite, and the color indicates the relative amounts of metabolites. Red indicates that the metabolite was expressed at high levels, whereas purple indicates lower levels. (**B**) Metabolite pathway enrichment analysis in the MCAOR group compared to that in the ShamR group. MCAOR, the ipsilateral (right) sides of brain tissues from the MCAO group; ShamR, the right sides of brain tissues from the Sham group (*n* = 10). The results were analyzed using Fisher’s exact test in R.

To further reveal metabolic alterations after AIS, we identified the top differential metabolites between the MCAOR and ShamR groups using hierarchical clustering analysis with heatmaps (Fig. 4A). We detected 20 metabolites, including moniliformin, octadec-6-enoylcarnitine, 2-methylbutyrolcarnitine, LysoPC (18:1(11Z)/ 0:0, LysoPS (18:0/0:0), and phenylacetylglycine, in the positive-ionization clusters and 17 metabolites, including L-aspartic acid, L-aspartyl-4-phosphate, N-alpha-acetyl-L-citrulline, arginyl-prolyl-proline, flavine adenosine dinucleotide, and oxidized glutathione, in the negative-ionization clusters. We then submitted these metabolites to perform KEGG pathway enrichment analysis and found that the following pathways were significantly altered in the MCAOR group compared with the ShamR group (*p* < 0.05): β-alanine metabolism, purine metabolism, glycine, serine, and threonine metabolism, cysteine and methionine metabolism, arginine biosynthesis, alanine, aspartate, and glutamate metabolism, and pantothenate and CoA biosynthesis (Fig. 4B). Most amino acid metabolic pathways mentioned above are intricately linked to the biosynthetic and homeostatic functions of mitochondria.

### ROS Accumulated in Both the Ipsilateral and Contralateral Sides of the Ischemic Brain

**Fig. 5:**
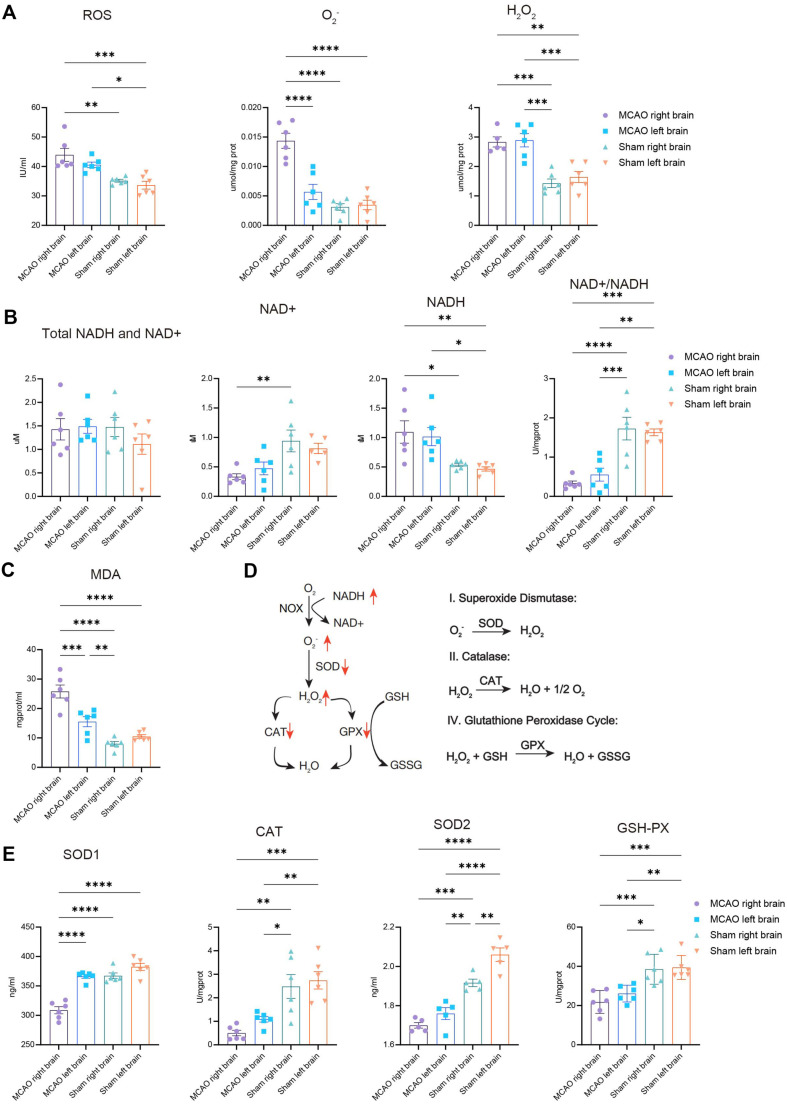
ROS accumulated in both ipsilateral and contralateral sides of the ischemic brain. (**A**) Levels of cerebral ROS, O_2_^-^, and H_2_O_2_ in the ipsilateral (right) and contralateral (left) sides of brain tissues from the sham and MCAO groups measured using ELISA. Total NADH and NAD^+^, NAD^+^, NADH, NAD^+^/NADH (**B**), and MDA (**C**) levels. (**D**) Graphic illustration of ROS production and elimination reactions: NOX transfers electrons to oxygen, leading to toxic accumulation of O_2_^-^ and NADH; SODs convert O_2_^-^ to H_2_O_2_, CAT in the cytosol or GPX in the mitochondria convert H_2_O_2_ to H_2_O and O_2_. (**E**) Levels of cerebral SOD1, SOD2, GPX, and CAT activity. Data are presented as the mean ± SD. Sham group versus the MCAO group. *n* = 6. One-way ANOVA and Tukey’s multiple-comparisons pos*t*-test were performed; *p < 0.05; **p < 0.01; ***p < 0.001, and ****p < 0.0001.

To validate our findings from the metabolomic analysis, we investigated the composition and metabolic functions of ROS in ipsilateral and contralateral brain tissues after AIS. We examined global brain ROS and the oxidants O_2_^-^ and H_2_O_2_. In the MCAO group, all three species increased in the ipsilateral hemisphere; O_2_^-^ levels were not significantly increased in the contralateral hemisphere (Fig. 5A). These data suggest that ROS production occurs rapidly in the early stages of AIS. Notably, the levels of NADH, a cofactor of NOX-mediated ROS production, and MDA, a highly toxic by-product of lipid peroxidation, markedly increased in the ipsilateral hemisphere (Fig. 5B and 5C). Altogether, our findings demonstrated that ROS accumulated in both the ipsilateral and contralateral hemispheres after ischemic lesions, whereas the infarcted ipsilateral hemisphere had a significantly higher ROS level.

We then probed the potential mechanisms underlying ROS accumulation and its role in AIS injury. First, we assessed the elimination of ROS in the cytosol and mitochondria (Fig. 5D). In the infarcted hemispheres of the MCAO-group animals, we observed impaired antioxidant and enzymatic defense systems in both the cytoplasm and mitochondria, as evidenced by the reduction of SOD1 and CAT in the cytosol and SOD2 and GPX in the mitochondria (Fig. 5E). These data demonstrate that in the infarcted core region, the accumulation of ROS may contribute to the impairment of the antioxidant and enzymatic defense systems in the cytosol and mitochondria.

In the contralateral hemispheres of MCAO-group animals, H_2_O_2_ elimination by the enzymatic defense system in the cytosol (CAT) and mitochondria (GPX) was reduced. The fact that accumulation of O_2_− occurs but is non-significant may be due to damage to the antioxidant system (SOD2) in mitochondria, which is partially rescued by SOD1 activity in the cytosol (Fig. 5E). In the contralateral hemisphere, ROS also accumulated during ischemia, but could be partially eliminated by antioxidant systems.

### ROS Accumulation may Trigger Mitophagy after AIS

**Fig. 6:**
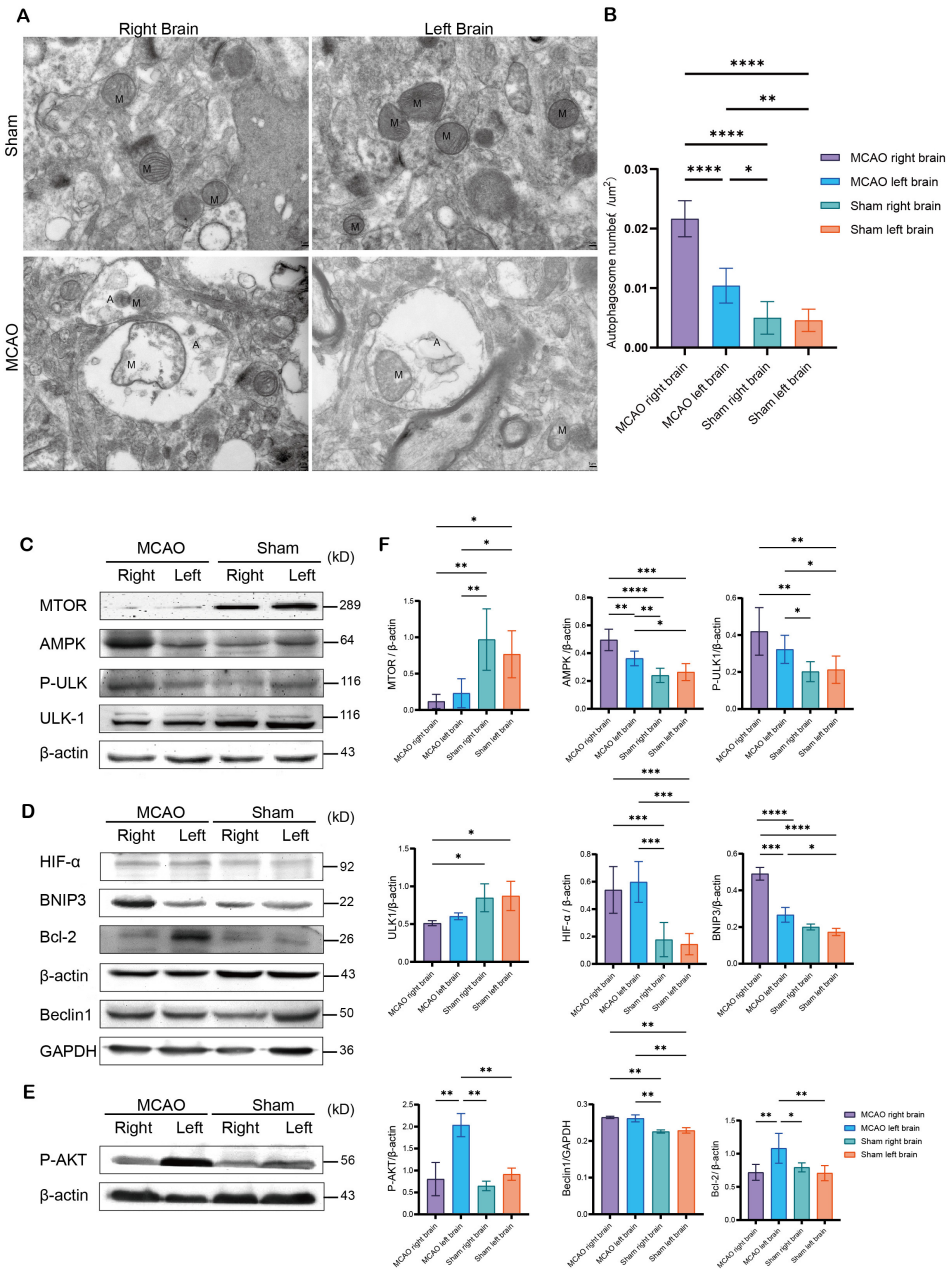
ROS accumulation may trigger mitophagy after AIS. Representative TEM images (**A**) and quantification (**B**) of mitophagosomes in the ipsilateral (right) and contralateral (left) sides of brain tissues from the MCAO group. Scale bar: 1 or 0.5 μm. Western blots (**C, D, E**) and quantification (**F**) of the protein levels of mTOR, AMPK, P-ULK1, ULK1, HIF-α, BNIP3, PAKT, Beclin1, and Bcl-2 in the ipsilateral (right) and contralateral (left) sides of brain tissues from the sham and MCAO groups. Quantification of band intensity after normalization to that of the loading control (β-actin or GAPDH). Data are presented as the mean ± SD. *n* = 6. Student’s *t*-test (**B**) or one-way ANOVA with Tukey’s multiple-comparisons pos*t*-test (**F**) was performed; *p < 0.05; **p < 0.01; ***p < 0.001; and ****p < 0.0001.

We further examined the function of mitochondria after ischemia and investigated how these pathways may be altered. We used TEM to examine mitochondria in MCAO animal brain tissues 36 h after MCAO. A higher number of autophagosomes containing mitochondria were observed in the infarcted ipsilateral region than in the contralateral hemisphere (Fig. 6A and 6B). These data suggest that the ipsilateral hemisphere has a higher level of mitophagy than the contralateral hemisphere after AIS.

Next, we examined the upstream signaling of mitophagy, including the mechanistic target of rapamycin kinase (mTOR) and adenosine 5’-monophosphate (AMP)-activated protein kinase (AMPK) pathways [21]. Western blot analysis showed decreased mTOR levels in both ipsilateral and contralateral hemispheres, and AMPK protein levels were significantly increased in the ipsilateral hemisphere (Fig. 6C). In mammals, autophagy is induced by the UNC-51-like kinase 1 (ULK1) complex. When AMPK is activated, the phosphorylated ULK1 complex forms the autophagesome [21]. We observed increased levels of p-ULK1 at ser556 in the ipsilateral and contralateral hemispheres (Fig. 6C). These results indicate that excessive ROS in the infarcted core may promote initial autophagosome formation through mTOR and AMPK signaling.

Bcl-2/adenovirus E1B 19 kDa interacting protein-3 (BNIP3) regulates autophagy by stimulating Beclin1 dissociation from Bcl-2 [22]. BNIP3 levels significantly increased in the ipsilateral hemisphere (Fig. 6D). We also observed significantly increased phosphorylation of AKT in the contralateral hemisphere (Fig. 6E). These data suggest that minor accumulation of ROS in the contralateral hemisphere may regulate mitophagy via p-AKT/mTOR signaling.

### AIS Causes Neuronal Apoptosis in the Ipsilateral but Not in the Contralateral Hemisphere

**Fig. 7:**
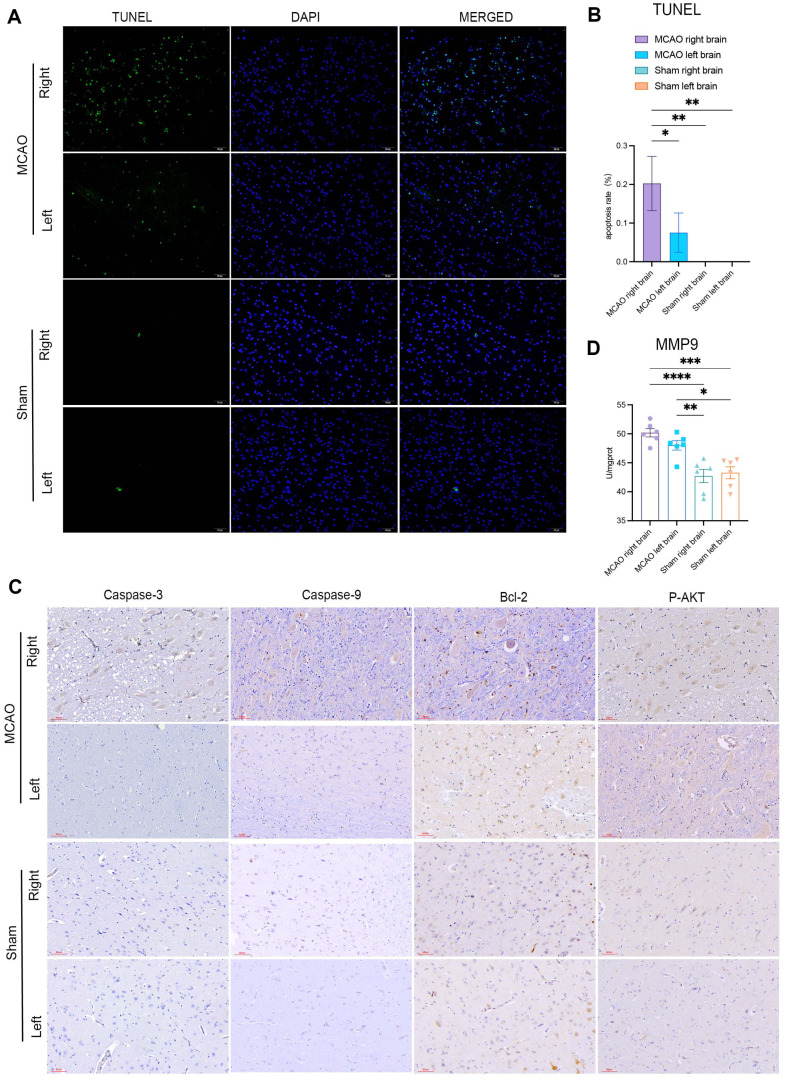
After AIS, excessive ROS may induce neuronal apoptosis in the ipsilateral but not the contralateral hemisphere. (**A**) Representative image of TUNEL (green), DAPI (blue), and merged staining of apoptosis; scale bar = 50 μm. (**B**) Quantification of apoptosis 36 h postoperatively in the MCAO group compared to the Sham group. (**C**) Immunohistochemical analysis of apoptosis markers, including caspase-3, caspase-9, Bcl-2, and p-AKT 36 h after stroke; scale bar = 60 μm. (**D**) Levels of cerebral MMP-9 were measured using ELISA. Data are expressed as the mean ± SD. The sham group versus the MCAO group; *n* = 6. One-way ANOVA and Tukey’s multiple-comparisons pos*t*-test (**B** and **D**) were performed; *p < 0.05; **p < 0.01; ***p < 0.001; and ****p < 0.0001.

Excessive ROS levels may induce apoptosis, as reflected by the markedly increased proportion of TUNEL-positive cells in the ipsilateral hemisphere (Fig. 7A and 7B). Next, we assessed the levels of apoptotic markers (caspase-3 and caspase-9) and anti-apoptotic proteins (Bcl-2 and p-AKT). In the ipsilateral hemisphere, caspase-3 and caspase-9 levels were increased in the cytoplasm (Fig. 7C), suggesting that impaired mitophagy may induce neuronal apoptosis upon ischemic dam. It was also found that MMP-9 accumulated in both hemispheres after MCAO (Fig. 7D).

In the contralateral hemisphere, the levels of Bcl-2 and p-AKT increased, suggesting that mild mitophagy might protect neurons from ischemic injury (Fig. 7C). Taken together, these results indicate that after AIS, activated caspase-9 induces apoptosome formation and activates caspase-3-mediated neuronal cell apoptosis, resulting in neuronal loss only in the infarcted hemisphere.

## Discussion

Given its high prevalence and devastating outcomes, ischemic stroke has attracted extensive research interest, particularly in the development of novel therapeutic strategies. However, only a few efforts have been successfully translated into clinical practice. A possible reason is that the vast majority of these studies were conducted in rodents, and the possible therapeutic effects do not translate to patients in large clinical trials [2]. Unlike humans, rodents have a gyral brain with a small range of white matter and 10% gray matter, whereas humans and pigs have similar proportions of white matter (exceeding 60%), which are considerably higher than those of mice and rats [23]. White matter ischemic injury plays a pivotal role in the prognosis of stroke and is the main driver of cerebral hemorrhage [24]. Thus, testing potential regimens in large animals, whose brains have anatomical and biological similarities with the human brain, has both scientific and translational significance. In the present study, we investigated the pathophysiological processes after ischemic stroke in minipigs while focusing on the potential role of ROS and dysfunctional mitophagy.

Our permanent electrocoagulation minipig model elegantly recapitulated the main pathophysiological features of ischemic lesions in the brain. For instance, MRI scans revealed an infarcted lesion in the frontal and temporal lobes, with extensive surrounding edema 36 h after MCAO surgery. The animals displayed severe hemiplegia, which is similar to clinical post-stroke symptoms in humans [3]. Thus, our model is superior to rodents in terms of phenotypic representation.

**Fig. 8:**
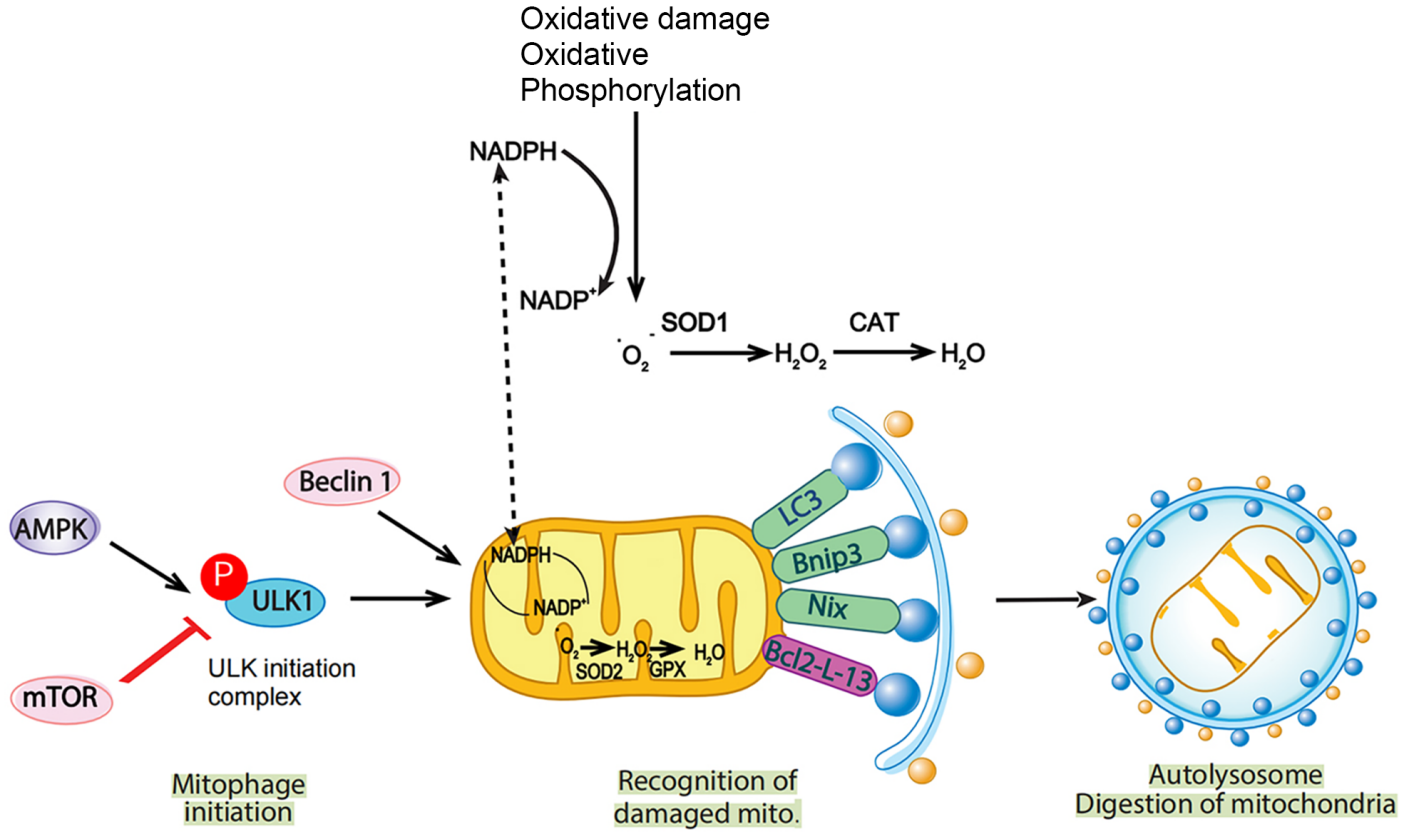
Schematic of the potential crosstalk between ROS and mitophagy. Excessive ROS levels in the ipsilateral hemisphere may be attributed to increased ROS production and impaired antioxidant (SOD1 and SOD2) and enzyme defense (CAT and GPX) systems, which may induce maladaptive mitophagy via the AMPK/mTOR-pULK-1-Beclin1 pathway.

In this study, we observed elevated ROS levels in both ipsilateral and contralateral hemispheres after AIS. Increased ROS levels may be attributed to increased production, suppressed elimination of ROS, or both (Fig. 8). We further examined the mechanisms underlying ROS accumulation. Notably, the ipsilateral hemisphere showed impaired antioxidant and enzyme defense systems, whereas the cytosolic antioxidant systems on the contralateral hemisphere were relatively intact. This may be partially explained by different ROS levels in the ipsilateral and contralateral hemispheres. By analyzing the metabolomic profiles of both hemispheres, we also proposed a functional association between amino acid metabolism and mitochondrial homeostasis in our minipig MCAO model.

Mitophagy is considered a protective strategy that may limit ROS production and promote cell survival under normal and mild stress conditions. However, excessive ROS can induce the overactivation of mitophagy (maladaptive mitophagy), which may induce cell death and tissue injury [25, 26]. We observed a high level of maladaptive mitophagy and neuronal apoptosis in the infarcted ipsilateral hemisphere; however, in the contralateral hemispheres, we observed a moderate level of mitophagy and no significant apoptosis. These data suggest that excessive ROS levels in the infarcted core after AIS may induce maladaptive mitophagy and neuronal apoptosis. Meanwhile, mild ROS levels in the contralateral hemisphere may induce adaptive mitophagy and promote neuronal survival.

One limitation of this study is that we only provided preliminary evidence regarding pathophysiological processes after ischemic stroke. Further mechanistic investigations would strengthen our suggestion that modulating excessive ROS-induced maladaptive mitophagy is a protective strategy for neurons. In addition to ROS accumulation, mitophagy can be triggered by various stressors. Further studies are warranted to gain a more comprehensive understanding of the mitophagy associated with ischemic lesions. Defining the cut-off threshold for adaptive and maladaptive mitophagy is also critical.

In conclusion, our study provides preliminary evidence of the accumulation of ROS after ischemic stroke and its potential association with maladaptive mitophagy and neuronal apoptosis in a minipig model. Our findings provide a basis for future studies investigating mitophagy modulators as potential therapeutic approaches for AIS.

## Supplemental Materials

Supplementary data for this paper are available on-line only at http://jmb.or.kr.



## Figures and Tables

**Table 1:** Heart rate, core body temperature, respiratory rate, and oxygen saturation of pigs during surgery.

Physiologic	Baseline	2 h	4 h	6 h	12 h	18 h	24 h	36 h
Heart rate (beats per minute)	69.00 ± 6.08	126.00 ± 15.13	90.67 ± 19.43	66.67 ± 29.30	84.33 ± 8.14	80.00 ± 25.16	90.67 ± 13.20	103.67 ± 17.62
Respiration (breaths per minute)	13.67 ± 1.53	22.67 ± 1.15	23.00 ± 2.65	19.67 ± 2.52	12.00 ± 2.65	12.00 ± 4.36	17.67 ± 5.51	11.33 ± 3.21
Blood oxygen saturation (%)	98.33 ± 0.58	87.00 ± 1.00	86.00 ± 4.36	93.67 ± .79	96.00 ± 3.46	96.00 ± 4.36	93.33 ± 2.89	94.67 ± 4.04
Temperature (°C)	38.70 ± 0.20	37.10 ± 0.35	38.97 ± 0.06	39.00 ± 0.10	39.17 ± 0.32	39.43 ± 0.12	39.97 ± 0.55	39.50 ± 0.44
