# Supplementary material for: Ischemic Stroke Induces ROS Accumulation, Maladaptive Mitophagy, and Neuronal Apoptosis in Minipigs
Source: J Microbiol Biotechnol. 2024 Nov 14;34(12):2648–61. doi: 10.4014/jmb.2409.09003 (PMC11729333; doi:10.4014/jmb.2409.09003)
Supplement: Supplementary file 1 [file jmb-34-12-2648-supple.pdf]

J. Microbiol. Biotechnol. 2024. 34(12): 2648–2661
https://doi.org/10.4014/jmb.2409.09003

Supplementary Tables

Table S1. Neurfunctional rating scale for the Miniature Pig.

	Description	Score
Appetite	All food consumed	0
	More than 50% food consumed	1
	50% food consumed	2
	Less than 50% food consumed	3
	No food consumed	4
Standing position	Normal standing position	0
	Unstable when standing	1
	Standing with support (leaning against wall)	2
	Stands up on stimulation	3
	Lying down: unresponsive to stimulation	4
	Immobile	5
Head position	Head erect	0
	Head raised on stimulation	1
	Unable to raise head	2
Utterance	Normal spontaneous vocalization	0
	Vocalizes on stimulation	1
	No vocalization	2
Gait	Walking with vitality	0
	Spontaneous unstable walking	1
	Unstable walking on stimulation	2
	Unable to walk	3
total		

Table S2. Motor functional score for the Miniature Pig.

	Description	Score
Forelimb	No motor disturbances	0
	Some contralateral weakness (instability during movement)	1
	Moderate contralateral paresis (instability on standing)	2
	Severe contralateral paresis	3
	Complete contralateral paralysis	4
Hindlimb	No motor disturbances	0
	Some contralateral weakness (instability during movement)	1
	Moderate contralateral paresis (instability on standing)	2
	Severe contralateral paresis	3
	Complete contralateral paralysis	4
Face	Symmetric (during rest and movement)	0
	Facial palsy	1
	Total	

Table S3. Antibodies and Elisa kit were used for this study.

Antibodies	Source	Catalog No.	Dilution
HIF-1 alpha	Bioss	bs-0737R	WB 1:500
ATG/ULK1	Bioss	bs-3602R	WB 1:300
phospho-ATG1(Ser556)	Bioss	bs-3464	WB 1:300
BNIP3	Bioss	bs-4239R	WB 1:500
Beclin1	Bioss	bs-1353R	WB 1:200
mTOR	Cell signaling technology	2983T	WB 1:1000
Bcl-2 alpha	Bioss	bs-15533R	WB 1:500
			IHC 1:100
Phospho-AKT(Thr308)	Bioss	bs-2720R	WB 1:300
			IHC 1:150
AMPK-alpha1	Bioss	bs-1115R	WB 1:300
GAPDH	Servicebio	GB15004	WB 1:3000
beta Actin	Servicebio	GB15003	WB 1:3000
Caspase-3	Cell signaling technology	9664T	IHC 1:100
Caspase-9	Cell signaling technology	9505T	IHC 1:100
ELISA KIT	Source	Catalog No.	
GPX-PX	Nanjing Jicheng	A005-1	
CAT	Nanjing Jicheng	A007-1-1	
O2-	Solarbio	BC1295	

MMP9	Mlbio	ml002342
ROS	Wuhan<br>Moshak Biotechnology	69-21611
TNF-β	Mlbio	ml002363
IL-10	Mlbio	ml002319
SOD1	Mlbio	YJ209403
SOD2	Mlbio	YJ249404
IL-1β	Mlbio	YJ002302
IL-6	Mlbio	YJ002311
MDA	Nanjing Jicheng	A003-1-2
SOD	Nanjing Jicheng	A001-3
NAD+/NADH	BEYOTIME	S0175
DAB Detection Kit	Gene tech	GK600705
